# A home-based comprehensive care model in patients with Multiple Sclerosis: A study pre-protocol

**DOI:** 10.12688/f1000research.7040.1

**Published:** 2015-09-18

**Authors:** Lufei Young, Kathleen Healey, Mary Charlton, Kendra Schmid, Rana Zabad, Rebecca Wester

**Affiliations:** 1 College of Nursing, University of Nebraska Medical Center, Lincoln, Nebraska, USA; 2 Multiple Sclerosis Program, Department of Neurological Sciences, College of Medicine, University of Nebraska Medical Center, Omaha, Nebraska, USA; 3 Department of Epidemiology, College of Public Health, University of Iowa, Iowa City, Iowa, USA; 4 Department of Biostatistics, College of Public Health, University of Nebraska Medical Center, Omaha, Nebraska, USA; 5 Department of Family Medicine, College of Medicine, University of Nebraska Medical Center, Omaha, Nebraska, USA

**Keywords:** multiple sclerosis, home-based care, disability, symptom management, healthcare utilization, value-based care

## Abstract

**Background **Disability is prevalent in individuals with multiple sclerosis (MS), leading to difficulty in care access, significant caregiver burden, immense challenges in self-care and great societal burden.  Without highly coordinated, competent and accessible care, individuals living with progressive MS experience psychological distress, poor quality of life, suffer from life-threatening complications, and have frequent but avoidable healthcare utilizations. Unfortunately, current healthcare delivery models present severe limitations in providing easily accessible, patient-centered, coordinated comprehensive care to those with progressive MS. We propose a home-based comprehensive care model (MAHA) to address the unmet needs, challenges, and avoidable complications in individuals with progressive MS with disabling disease.

**Objective **The article aims to describe the study design and methods used to implement and evaluate the proposed intervention.

**Method** The study will use a randomized controlled design to evaluate the feasibility of providing a 24-month, home-based, patient-centered comprehensive care program to improve quality of life, reduce complications and healthcare utilizations overtime (quarterly) for 24 months. A transdisciplinary team led by a MS-Comprehensivist will carry out this project. Fifty MS patients will be randomly assigned to the intervention and usual care program using block randomization procedures. We hypothesize that patients in the intervention group will have fewer complications, higher quality of life, greater satisfaction with care, and reduced healthcare utilization. The proposed project is also expected to be financially sustainable in fee-for-service models but best suited for and gain financial success in valued-based care systems.

**Discussion** This is the first study to examine the feasibility and effectiveness of a home-based comprehensive care management program in MS patients living with progressive disability. If successful, it will have far-reaching implications in research, education and practice in terms of providing high quality but affordable care to population living with severe complex, disabling conditions.

## Background

While great strides have been made in the treatment of relapsing forms of multiple sclerosis (MS), many individuals have or will enter a progressive phase of this disease. This phase of disease is dynamic, highly complex and disabling, which presents extensive challenges in all aspects of care delivery ^1, 2^. The progressive phase of MS meets criteria for the description of “a consuming illness” outlined by the Patient-Centered Medical Home Model (PCMH) ^3^. Specific problems and rate of progression vary, but many will have dysfunction in gait ^4, 5^, movement of extremities ^4^, bladder and bowel function ^6, 7^, speech and swallowing ^8^, and respiratory musculature ^9, 10^. Over half will have physical pain ^11^, generally related to neuropathy and/or spasticity ^12^. Cognitive dysfunction ^13^ and depressive mood disorders ^14– 17^ are prevalent. Family members are also likely to suffer from caregiver burden ^18^, mood disorders ^19^ and strain on their own health ^18^. As the disease progresses, a significant proportion of patients will need assistive devices, including power mobility, urinary catheters, gastric tubes, hospital beds, home modifications, and other devices ^1, 2^. Complications can be life threatening, including falls ^20, 21^, urinary tract infections (UTIs) ^22^, respiratory conditions (pneumonia and influenza) ^23, 24^, and pressure ulcers ^21^. Consequently, patients’ health related quality of life decreases substantially as disability ensues ^25^. The MS Society’s White Paper captures the cry of those with progressive MS, who feel “disconnected,” “underserved,” “isolated,” “forgotten,” and “overwhelmed” ^26^. Patients ultimately face the loss of independence in virtually all aspects of life. Still, families desire to keep their loved one at home when possible but acknowledge the overwhelming impact of the disease on the entire family ^27^. Moreover, due to the limitations in mobility, cognition and communication, access to care is highly challenging ^28, 29^. As a result, patients living with progressive phase MS have numerous complex and dynamic healthcare needs that require a range of primary care, specialty, multidisciplinary, and community resources for a long period of time (their life expectancy) ^30, 31^. However, the current care delivery and payment systems contribute barriers and challenges in providing comprehensive coordinated care ^32^, leading to unnecessary healthcare utilization and delayed effective treatments ^33^. Without the comprehensive disease management by a designated provider, the quality of care received is often suboptimal or poor due to the fragmented care system in which the acute care based providers are unfamiliar with patients’ needs, have little knowledge and experience caring for patients with MS ^34^. The combination of the current ambulatory care system utilizes a fee-for-service payment model combined with shortage of MS care specialists leaves no time to address complex chronic care issues or advance care planning ^35– 37^.

To address these problems and gaps in inadequately caring for patients living with progressive MS, we propose a home-based, patient-centered, comprehensive care management program led by a ‘MS-Comprehensivist’. The program is designed to provide a full range of medical and social services for patients and their caregivers, including a transdisciplinary team of primary care providers, specialists, care managers, rehabilitative, social home health, and personal care services. The MS-Comprehensivist who is an advance practice nurse specialized in MS care is responsible to 1) make regular house calls to address patients/family specific needs, 2) coordinate the care with the primary and specialty providers, 3) identify and mobilize other community resources, and 4) provide staff training and patient education in co-managing the complex complications and symptoms. This program, referred to as **M**ultiple Sclerosis **A**t **H**ome **A**ccess (MAHA), incorporates core principles of the chronic care and patient-centered medical home models. The purpose of the proposed study is to examine the feasibility (e.g., acceptability, utility, implementation, financial sustainability, adaptation and integration) ^38^ and effect of the MAHA model on patient-centered outcomes (i.e., complications, quality of life, satisfaction) and health care utilization outcomes (i.e., unplanned hospitalization and ED visits). The following specific aims were designed to achieve this purpose.

**Specific Aim 1**: To evaluate the effect of the MAHA model on 1) the numbers of complications; 2) patients’ quality of life; and 3) patients’ satisfaction over time (baseline and quarterly).

**Specific Aim 2**: To evaluate the effect of the MAHA model on 1) the numbers of emergency room (ER) visits, and 2) the numbers of unplanned hospitalizations.

**Specific Aim 3**: To evaluate the feasibility of the MAHA model.

1) Acceptability by assessing patient and family experience with the program, as well as provider satisfaction
2) Utility by examining the actual use of the program and number of referral received by other providers
3) Implementation by evaluating the amount and type of resources needed to implement and factors affecting implementation;
4) Adaptation by evaluating the selected elements of the program delivered by tele-health is as effective as face-to-face format.
5) Financial sustainability and cost saving by comparing the estimated total cost of the program and financial compensation from payers.

## Conceptual framework

**Figure 1.**
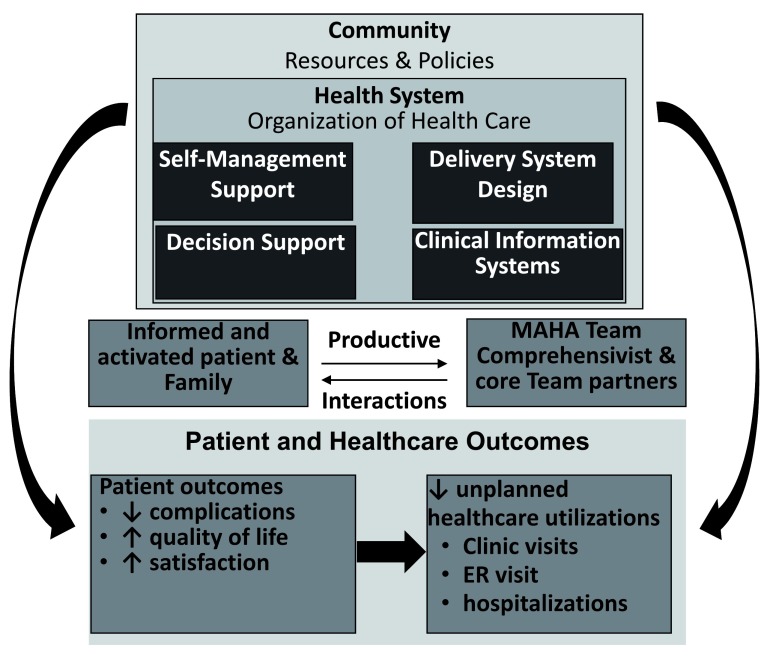
MAHA Model Framework adapted from Wagner’s Chronic Care Model.

The conceptual framework supporting the MAHA program ( Figure 1) is designed based on the core elements in Wagner’s Chronic Care Model (CCM) and Donabedian’s Structure-Process-Outcome (SPO) model ^39^. First, based on Wagner’s CCM, the MAHA model emphasizes the re-design of existing community and healthcare systems to be patient-centered ^40^. From the patient-provider level, the unique MAHA model again is structured surrounding patients’ needs with the following key components: 1) the productive interaction between informed, activated patient/family and the MAHA team; 2) a transdisciplinary team led by an MS-Comprehensivist who is an advance practice nurse with expertise in MS care, chronic disease management and primary care; 3) care coordination and effective communication among care team members. As a result of re-designing care systems and processes, it is expected that patient outcomes (e.g, complication, quality of life and satisfaction of care) are improved, leading to reduced unplanned healthcare utilizations.

Besides its function as an acronym for **M**ultiple Sclerosis **A**t **H**ome **A**ccess, the term **MAHA** is derived from its city of origin in Omaha, Nebraska, which was settled by Native Americans of the Omaha tribe. In their language, Omaha means “against the wind or current,” which reflects American Indians survival experience from severe weather, disease and scarcity ^41^. While caring for and supporting those MS patients with profound disabling and chronic, complex complications, the caregivers and providers often feel overwhelmed by the environmental barriers, much like the Omaha tribe must have felt centuries ago. Consistent with the core values of these original settlers (earth and sky) ^41^, we honor holistic care which includes evidence-based medicine (earth) and equally holistic care including social, emotional, and spiritual applications (sky).

## Methods

### Study design

The study is a prospective, two-group, randomized experimental design with nine data collection points (baseline and every three months). MS patients recruited from a neurology clinic will be randomized into two groups: the intervention or usual care group. The usual care group receives the current standardized MS care, while the intervention group receives usual care plus 24-months of the MAHA intervention provided by a transdisciplinary team led by a MS-Comprehensivist. The usual care group will receive the intervention at the end of the 24-month period if the intervention is found to be effective by our a priori criteria. The study is subject to review and approval by the University of Nebraska Medical Center Institutional Review Board (IRB) and informed consent will be obtained from all participants prior to the study.

### Study settings

Potential subjects will be identified and recruited from the University of Nebraska Medical Center (UNMC) neurology clinic, where the principle investigator, who has ethical access at the clinic site, will be responsible for identifying the potential participants, screening for eligibility and referring eligible subjects for recruitment.

### Study participants

***Inclusion criteria.*** Patients are eligible for the study if they: 1) have a diagnosis of progressive MS; 2) receive a Kurtzke Expanded Disability Status Scale (EDSS) score ≥6.5 (requires bilateral assist for ambulation and cannot walk > 120 meters; http://www.nationalmssociety.org/NationalMSSociety/media/MSNationalFiles/Brochures/10-2-3-29-EDSS_Form.pdf); and 3) have a home residence located within 60 miles of the Omaha metropolitan area.

### Sample size

The sample size estimation is computed based on **Specific Aim 1**: the increased score of quality of life (QoL) and **Specific Aim 2**: the reduced number of unplanned healthcare utilizations associated with complications. An estimated 50 patients per group will provide 80% power to detect a 5-point difference [Usual Care mean (SD): 45(8.6), MAHA mean (SD) 50(8.6)] between groups in the SF-36 Mental Health Component Score QoL. This sample size also provides 80% power at a 5% significance level to detect a 34% reduction in unplanned visits/admits (from 2.5 per person per year to 1.65, using the Poisson distribution, which is most appropriate for count/rate data). These are feasible and clinically important differences. Based on our preliminary analyses, 125 patients are currently expected to meet these criteria. Furthermore, based on our previous patient survey results, a high proportion of patients (~80% or n = 100) have expressed a genuine interest in participating in MS research for the benefit of others with this disabling disease. Furthermore, we estimate 50 patients will be a reasonable panel size for the MS-Comprehensivist or care team leader (CTL) in the MAHA model (unpublished report).

### MAHA intervention

The MAHA model is a patient- and family-centered system in which care is tailored around the complex, chronic needs of those with progressive disabling MS. The intervention program is designed to address a fundamental question: “how will this affect the patient and/or family?” The majority of care and medical services will be provided at home, thus avoiding frequent and cumbersome clinic visits. The intervention strategies were developed based on the frequent requests and suggestions from patients with progressive MS, along with caregivers and providers experiencing daily struggles with fragmented care. Furthermore, the model is also supported by initiatives from the health policy literature developed by the National Multiple Sclerosis Society (NMSS), emphasizing the need for home- and community-based services ( http://www.nationalmssociety.org/Treating-MS/Comprehensive-Care).

**Figure 2.**
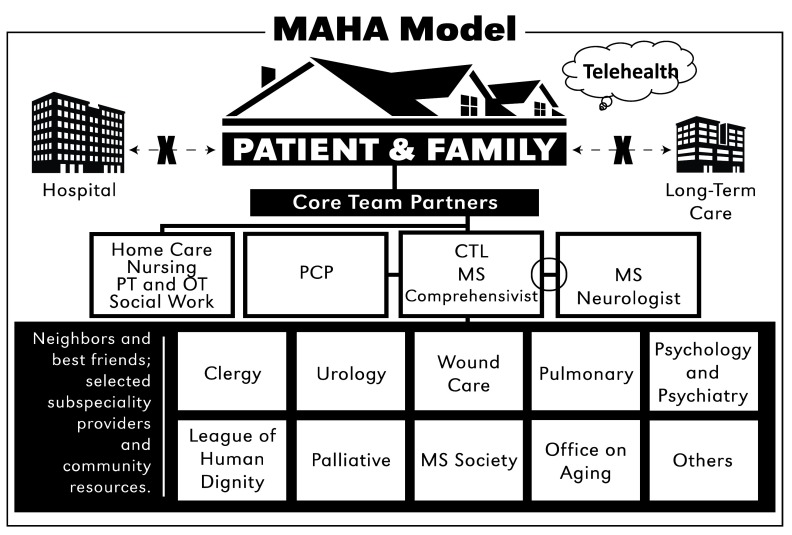
MAHA Model.

***MAHA Team Structure.*** The intervention will be delivered by a transdisciplinary team led by a MS-Comprehensivist, also referred to as Care Team Leader (CTL). The care provided is comprehensive and holistic, addressing patient/family physical, emotional and spiritual, and social needs. The transdisciplinary team includes the core team partners and “neighbors and best friends” ( Figure 2). The CTL role will be filled by a nurse practitioner (NP) with specialty training in MS and extensive experience managing patients with chronic illness. The core team partners include the CTL, MS neurologist, primary care provider (PCP), and selected home health agencies. To address MS patients’ complex needs and reduce unplanned healthcare services, the CTL will closely collaborate with patients and family, the MS neurologist and the PCP to develop patient-centered care plans and goals, and to co-manage symptoms and complications. The selected home health agencies will provide nursing and personal care staff, physical and occupational therapists (PT/OT), and social work services. They are responsible for carrying out the care plan and conducting collaborative, on-going evaluation of the care plan with the CTL. Given MS patients’ limitations in mobility, PT/OTs will also be involved in plans of care to facilitate the restoration and maintenance of function oriented toward activities of daily living (ADLs). PTs from the home health agencies will be trained by physical therapists who have extensive experience in the education and care of MS patients. “Neighbors & Best Friends” represent subspecialty providers and community resources. The “neighbors”, or subspecialty providers (e.g., urologist, rehabilitative professionals, palliative care specialist, ophthalmologist, wound care specialist, etc.) provide consultation services and advise the CTL in preventative and treatment strategies for common complications or issues not responsive to standard strategies. The “Best Friends”, include community resources, including clergy, respite services, the MS Society, League of Human Dignity, and Office on Aging, among others.

**Figure 3.**
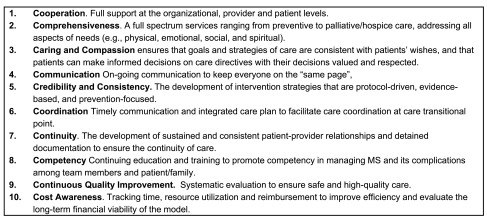
Ten fundamental elements guiding the care process.

***MAHA Team Processes (10 C’s).*** The MS-Comprehensivist or CTL will be responsible to: 1) provide regular and frequent visits to patients’ homes using a pre-planned schedule based on each individual’s care needs; 2) coordinate patients’ care by collaborating and communicating closely with the core team partners, and “neighbors and best friends”; and 3) train the home health staff in the specialty care of MS patients. In the process of care, the MAHA model stresses patient/family engagement (i.e., informed and activated) and productive interaction between patients and providers through ten fundamental elements (10 Cs; Figure 3).

### Data collection, instruments and sources of data

Key data collection instruments, assessments, and measures are summarized in Table 1. This information will be collected from three data sources: 1) patient reports to CRC personnel, 2) patient reports to the CTL or other MAHA team member at each home visit, and 3) medical record review.

**Table 1.** Study measures.

Data Collection Instrument/Assessment		Method, Location, Data Source	Measure Timeline (in months)								
			BL*	3	6	9	12	15	18	21	24
Kurtzke Expanded Disability Scale (EDSS)		Neurologist/CTL to assess in person	X				X				X
PATIENT REPORTED MEASURES											
-Demographics		CRC to collect at baseline visit and via mail/phone	X								
-Functional activity of daily livings (ADLs) (Barthel) ^42^			X	X	X	X	X	X	X	X	X
-Multiple Sclerosis QoL Inventory (MSQLI) ^43^			X				X				X
-Novak Caregiver Burden Inventory (CBI) ^44^			X	X	X	X	X	X	X	X	X
-Client Satisfaction Questionnaire (CSQ-8) ^45^			X		X		X		X		X
POTENTIAL COMPLICATONS											
-Pneumonia -Urinary Tract Infection (UTI)	-Falls -Pressure Ulcer	CRC to collect at baseline visit, via mail/ phone and from medical record review. Providers assess during in-person visits	X	X	X	X	X	X	X	X	X
HEALTH SERVICES USE											
Emergency Room (ER) -Unplanned (acute)	Inpatient Admissions -Planned (elective) -Unplanned (acute) -Length of Stay	CRC to collect at baseline visit and via mail/phone. Providers assess during in-person visits. Data are retrieved from medical record.	X	X	X	X	X	X	X	X	X
CPT Codes and Reimbursement Amounts		UNMC Neurology Clinic Database	X		X		X		X		X
MAHA RESOURCE INTENSITY											
-Home visit time logs -On-call activity logs -Coordination care management activities		MAHA staff to document after each home visit and on-call activity	X	X	X	X	X	X	X	X	X

*Baseline questionnaires will be performed at the baseline clinic visit

### Data analysis

***Specific Aim 1 and 2.*** The data analysis will follow the intention-to-treat (ITT) protocol. To ensure groups are comparable, descriptive analyses, including t-tests and Chi-square tests will be conducted to compare the characteristics of each group at baseline. The occurrence of complications and other self-reported measures, such as ADLs, Multiple Sclerosis QoL Inventory (MSQLI), CSQ-8, and Caregiver Burden Inventory (CBI) will be compared between groups using a linear mixed model to account for the repeated measurements over time and adjust for baseline scores of the respective measures. To determine if differences exist between groups after adjusting for potential confounding variables, such as age, gender, comorbidity, etc. or imbalances between groups, unplanned healthcare utilization (i.e., visits/admissions) will be analyzed using Poisson regression models. The proportion of any versus no use of each health service (e.g., proportion with any inpatient admissions versus the proportion with no admissions) will be compared between groups using Chi-square tests. The time-by-group interaction will be investigated to determine if any changes over time are consistent between groups.

***Specific Aim 3.*** To assess the acceptability, utility, implementation and adaptation of the program, we will create a toolkit that comprehensively describes our program structure, processes, patients/provider/system outcomes, as well as lessons learned. This toolkit will serve to evaluate the MAHA program and identify system-, provider-, and patient-levels of barriers and facilitators. To evaluate financial sustainability, we will specifically track the Current Procedural Terminology (CPT) codes for evaluation and management visits (clinic and home visits), care coordination, and care plan oversight. Reimbursement amounts associated with these codes will be summed for each study group at six and 12 months. T-tests and log transformed linear regression models will be used to compare total reimbursement amounts between groups. Also, staffing and associated salary estimates will be computed based on home visit time logs maintained by the MAHA team members, on-call activity logs, and number of home visits in the intervention group. We will project the necessary percent FTE and salary to execute the intervention model based on a range of panel sizes (e.g., 25, 50, 100 patients) per MS-Comprehensivist/CTL. The ratio of total salary costs-to-reimbursement amount for a given number of patients will be calculated for each group and compared using t-tests.

## Discussion

The study is highly relevant to MS patients overwhelmed by the challenges of accessing healthcare and the myriad of complications related to such a progressive, disabling disease. The purpose is to examine the feasibility and effectiveness of a home-based, patient-centered, comprehensive care model on patient reported and healthcare utilization outcomes. Although the tools, expertise, and strategies to prevent many of these complications and to improve quality of life exist and prove to be effective, their use and implementation is hindered by a siloed system, fragmented care processes, and an inappropriate volume-based payment system. The proposed study is designed to evaluate an innovative model to meet the complex needs and overcome the self-care challenges of those MS patients in a progressive, disabling stage of disease.

**Implication to Practice:** The study findings and all project materials will assist other institutions and providers in adapting the MAHA model to manage MS and other populations living with chronic complex conditions. In addition, the study findings may inform NMSS to develop and/or update guidelines in managing MS patients in progressive stage, leading to the improved care and prolonged lives of MS patients.

**Implication to Education**: The toolkits and manuals we developed for patient/provider education could be integrated into the curriculum in various healthcare professional programs and modified to be continuing education packets for clinicians.

If proven successful, we will further adapt the model components and intervention strategies to be delivered via telehealth to reach rural/remote populations facing significant challenges in accessing care. Based on our preliminary analysis and the expertise of our team, we believe the MAHA intervention will be the missing ingredient for mitigating the challenges in managing populations living with chronic and complex illness. The long-term goal of this study is to conduct a larger scale study and create a template of chronic complex disease management to be expanded and disseminated in managing all population living with disabling and consuming conditions.
