# Supplementary material for: A home-based comprehensive care model in patients with Multiple Sclerosis: A study pre-protocol
Source: F1000Res. 2015 Sep 18;4:872. [Version 1] doi: 10.12688/f1000research.7040.1 (PMC4670009; doi:10.12688/f1000research.7040.1)
Supplement: Peer review history

# Peer review: Referee response for version 1

**DOI:** 10.5256/f1000research.7578.r11257

**Published:** 2015-12-02

**Authors:** M. Rashad Massoud, Amanda Ottosson

**Affiliations:** 1 Department of Public Health, University Research Co LLC, Bethesda, MD, USA; 2 University Research Co LLC, Bethesda, MD, USA

This is an important study and is very well designed. The study has a clear objectives and aims to overcome four challenges in care for patients with multiple sclerosis. The objective of the article is to describe the study design and methods that were used to implement and evaluate the use of the home-based comprehensive care model (MAHA) to overcome the following four challenges:

1. Difficulty in care access
2. Caregiver burden
3. Self-care challenges
4. Societal burden

The intervention uses the MAHA and Chronic Care Model. 50 patients will be randomly assigned to receive the intervention or usual care. The authors hypothesized that there would be fewer complications, higher quality of life, greater satisfaction with care, and reduced healthcare utilization to patients who received the intervention.

The authors described the theory of change well and it is plausible. We have one suggestion to the investigation. If the objective is to improve care in patients with progressive multiple sclerosis, then iterative testing of changes, and learning from these tests, may be more valuable than testing the effectiveness of a "set intervention".

This type of inquiry might lend itself to an ongoing type of treatment and modifications of the implementation of the intervention as opposed to an endline assessment. This would allow for a deeper understanding of the elements of the intervention that did and did not work. More importantly, one could look into why and how they worked or did not.

The title and the abstract are appropriate for the article. We look forward to seeing the results of the study.

We have read this submission. We believe that we have an appropriate level of expertise to confirm that it is of an acceptable scientific standard.

# Peer review: Referee response for version 1

**DOI:** 10.5256/f1000research.7578.r10803

**Published:** 2015-10-23

**Authors:** Anisha Doshi, Jeremy Chataway

**Affiliations:** 1 The Queen Square Multiple Sclerosis Centre, University College London, London, UK; 2 Queen Square Multiple Sclerosis Centre, University College London, London, UK

This article aims to define a new study pre-protocol examining the efficacy of a home-based comprehensive care model, Multiple Sclerosis At Home Access (MAHA), in patients with Progressive Multiple Sclerosis (MS) versus the current healthcare delivery models. In particular the study pre-protocol describes a prospective 24 month block randomised controlled model using an estimated cohort of 50 patients in each arm with the following inclusion criteria; Progressive MS, Kurtze EDSS above or equal  to 6.5,and live in a residence within 60 miles of the Omaha metropolitan area. The study is subject to review and approval by the University of Nebraska Medical Center Institutional Review Board (IRB). It would be useful to outline the currently available comprehensive care model. The proposed study measures and data analysis should be sufficient in answering the outcome measures and specific study aims.

In our opinion, this study pre-protocol, if approved, will add to the already established data with regard to comprehensive care models, and in particular may be of relevance to those focussing on patients with significant disability due MS as well as to other conditions.

References:

PMID 23692584

PMID 22247419

PMID 20047364

We have read this submission. We believe that we have an appropriate level of expertise to confirm that it is of an acceptable scientific standard.
